# Left renal vein thrombosis secondary to compression by the uncinate process of the pancreas, mimicking the nutcracker syndrome

**DOI:** 10.1590/0100-3984.2015.0192

**Published:** 2017

**Authors:** Rodolfo Mendes Queiroz, Daniel de Paula Garcia, Mauro José Brandão da Costa, Eduardo Miguel Febronio

**Affiliations:** 1 Documenta – Hospital São Francisco, Ribeirão Preto, SP, Brazil.

**Figure 1:**
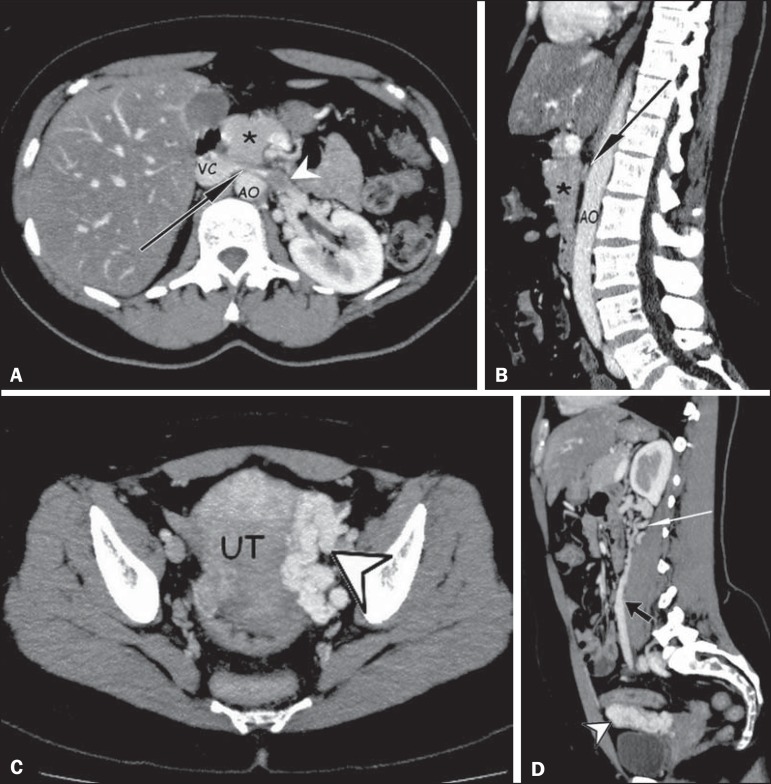
**A,B:** Contrast-enhanced arterial-phase CT of the abdomen, in the axial plane and in sagittal reconstruction, respectively, characterizing clots within the proximal portion of the LRV (white arrowhead), showing a reduction in its caliber at the aortomesenteric compression (black arrows), close to its junction with the inferior vena cava (VC), due to the extrinsic compression exerted by the uncinate process of the pancreas (asterisks) against the aorta (AO). Additional finding: diffuse hypointense signal in the hepatic parenchyma, suggesting fatty infiltration. **C,D:** Contrast-enhanced arterial-phase CT of the abdomen, in the axial plane and in sagittal reconstruction, respectively, showing dilation of the pelvic vessels (arrowheads) near the left lateral aspect of the uterus and the left gonadal vein (black arrow), with dilated collateral veins in the ipsilateral perirenal space (white arrow).

*Dear Editor*,

A 40-year-old woman presented with a five-month history of pain in a portion of the mesogastrium and in the left side. She reported recent weight loss (of 5 kg) after having had dengue fever. She also reported no comorbidities and stated that she was not using contraceptives. The physical examination revealed Giordano’s sign on the left side. The results of the blood count and urinalysis were normal. Computerized tomography of the abdomen showed compression of the left renal vein (LRV) caused by the uncinate process of the pancreas pressing against the aorta, leading to dilation of the proximal segment, with an intraluminal thrombus (Figures 1A and 1B), dilation of the collateral perirenal venous system, dilation of the gonadal veins, and ipsilateral pelvic varices (Figures 1C and 1D). The patient was treated with oral anticoagulants for four months and declined to have a stent placed in the LRV.

Vascular compressive syndromes occur in less than 1% of the cases and represent vascular trapping between rigid surfaces, which lead to manifestations caused by hypertension, venous congestion, thrombosis, and arterial ischemia^(1-4)^.

The causes of compression of the LRV include expansive retroperitoneal formations, anatomical variations, and nutcracker syndrome (NCS)^(2)^. NCS is usually caused by the trapping of the LRV between the superior mesenteric artery and the abdominal aorta (aortomesenteric compression)^(1-5)^. In rare cases, the LRV is retroaortic. In such cases, compression occurring between the aorta and the spine is known as posterior NCS^(2-4)^. The nutcracker phenomenon corresponds to these findings without clinical correlation ^(2-4)^. The prevalence of NCS is unknown, although it is known that it occurs predominantly in healthy, thin individuals between 20 and 40 years of age and in women^(1-4)^. Clinically, hematuria is the most common finding, followed by pain on the left side, dyspareunia, dysmenorrhea, dysuria, varicoceles, and pelvic varices^(1-5)^. In exceptionally rare cases, anatomic variations in the pancreas compress nearby vessels, including the LRV^(2,6,7)^.

Renal vein thrombosis (RVT) is common in nephrotic syndrome and in severely hypotensive neonates. Other causes: traumas, surgery, infections, neoplasias, vasculitis, venous compressions, contraceptives and myeloproliferative diseases. It’s infrequent in healthy adults, predominantly unilaterally^(8,9)^. The clinical presentation of RVT is much like that of NCS, with the added features of an acute increase in renal volume, late atrophy, and progressive deterioration of renal function, as well as the complication of pulmonary thromboembolism in up to 50% of cases^(5,8,9)^.

The pathophysiology of thromboses encompasses Virchow’s triad: endothelial lesions, stasis, and hypercoagulability. Generally, thrombotic events involve at least two factors, although one may be sufficient^(5,8,9)^.

One of the principal methods employed in the diagnosis of NCS is Doppler ultrasound, which is noninvasive and can be used in determining venous caliber and flow, the latter being suggestive of NCS when it exceeds 100 cm/s, with a sensitivity and specificity of 78% and 100%, respectively, for the diagnosis^(1-4)^. It shows high sensitivity in the investigation of RVT^(8)^. Ultrasound, however, is operator-dependent and may not detect small thromboses^(8,9)^. For the diagnosis of NCS and RVT, angiography has a sensitivity of 66.7–100% and a specificity of 55.6–100%^(8)^. It is able to evaluate the aortomesenteric angle (compression); possible compression and dilation of the LRV; filling defects; endoluminal blood clots; and signs of chronic thrombosis, such as thickening of the vessel walls and calcifications^(1-4,9)^. However, it uses radiation and potentially nephrotoxic contrast agents^(8,9)^. Retrograde venography is the gold standard examination in NCS^(1-4)^ and RVT^(8)^; it shows pressure gradients greater than 3 mmHg in the LRV, in addition to the filling defects that represent thrombi^(1-4,8)^. However, it is invasive, potentially triggering thrombosis, and uses intravenous iodine^(8)^.

The therapeutic options are conservative treatment, reimplantation/ transposition of the LRV, the use of an external or internal stent, renal autotransplantation, gonadocaval bypass, and nephrectomy^(1-5)^. If RVT occurs, anticoagulation and thrombolysis can also be employed^(8-10)^.
